# Alterations of Spontaneous Brain Activity in Hemodialysis Patients

**DOI:** 10.3389/fnhum.2020.00278

**Published:** 2020-07-15

**Authors:** Hui Juan Chen, Jie Qiu, Qingqing Fu, Feng Chen

**Affiliations:** ^1^Department of Radiology, Hainan General Hospital (Hainan Affiliated Hospital of Hainan Medical University), Haikou, China; ^2^Department of Ultrasound, Hainan General Hospital (Hainan Affiliated Hospital of Hainan Medical University), Haikou, China; ^3^Department of Radiology, Hainan General Hospital (Hainan Affiliated Hospital of University of South China), Haikou, China

**Keywords:** resting-state fMRI, amplitude of low-frequency fluctuation, end-stage renal disease, hemodialysis, default mode network

## Abstract

Cognitive impairment is prevalent in hemodialysis end-stage renal disease (ESRD) patients. It might be associated with poor prognosis. Nevertheless, the underlying mechanisms have not been completely clarified. This study explored spontaneous brain activity in ESRD patients on hemodialysis by using the amplitude of low-frequency fluctuation (ALFF). Nineteen ESRD patients on regular hemodialysis were included in this study. Eighteen age-, sex- and education level-matched volunteers were enrolled as the healthy control group. All participants had resting-state functional MRI scanning, neuropsychological tests, and laboratory testing. ALFF was used for assessing intrinsic brain activity. Independent samples *t*-test was used for obtaining group differences. Spearman correlation analysis was applied to assess the association between ALFF changes, neuropsychological, and clinical indices. Compared to the healthy control group, hemodialysis patients showed decreased ALFF in the precuneus, right angular gyrus/inferior lobule as well as increased ALFF in the left parahippocampus/hippocampus and right precentral/postcentral gyrus. The ALFF abnormalities in these regions were closely associated with hemoglobin levels. Also, increased ALFF in the left parahippocampus/hippocampus showed a negative correlation with the score of long-delayed free recall. Hemodialysis patients had aberrant ALFF in the default mode network (DMN) regions, particularly in the precuneus and parahippocampus/hippocampus, which may be correlated with neuropathological mechanisms involved in hemodialysis.

## Introduction

Cognitive dysfunction commonly occurred in patients with end-stage renal disease (ESRD; Murray et al., 2006; Drew et al., 2017). This is specifically high in those treated with hemodialysis. It is reported that approximately 70% of patients on hemodialysis who are aged no less than 55 years old have moderate to severe cognition dysfunction, which was four times equal to age-matched controls (O’Lone et al., 2016). Cognitive dysfunction includes memory decline, attention distraction, and diminished motor ability. ESRD patients usually had a high level of depression and anxiety. Importantly, cognitive impairment is independently correlated with lower quality of life, the risk for hospitalization, higher rates of morbidity and mortality (Griva et al., 2010), indicating an urgent need to explore the pathophysiological mechanism of cognition impairments in hemodialysis ESRD patients.

Resting-state functional MRI has been accepted as an effective method to evaluate the neuropathological mechanisms of cognition impairment in ESRD patients (Liang et al., 2013; Ni et al., 2014; Zhang et al., 2016). Functional neurologic imaging studies demonstrated reproducible findings of abnormalities in the default mode network (DMN) regions in ESRD patients (Liang et al., 2013; Ni et al., 2014). The posterior cingulate cortex, precuneus, medial prefrontal cortex, and inferior parietal lobule are the main components of the DMN. However, as far as we are concerned, only several resting-state functional MR imaging studies (Li et al., 2014, 2018; Shi et al., 2019) have been performed in hemodialysis ESRD patients. Li et al. (2014) demonstrated that normal regional homogeneity change was mainly found in the memory and cognition associated cortical regions in ESRD patients. By using the functional connectivity density, Shi et al. (2019) revealed abnormal intrinsic dysconnectivity pattern of salience network-associated regions in hemodialysis ESRD patients from the whole-brain network perspective. Zang et al. (2007) developed the amplitude of low-frequency fluctuation (ALFF), which can depict the intensity of amplitude alterations of blood oxygen level-dependent signal. ALFF has been regarded as an effective tool to measure the intensity of spontaneous brain alteration (Liu et al., 2013). It has been widely accepted in various mental disorders such as Alzheimer’s disease (Yang et al., 2018, 2019) and schizophrenia (Wang et al., 2019). The influence of the hemodialysis on intrinsic regional brain activity in ESRD patients has not yet been fully explored, because most of the previous studies included hemodialysis patients, peritoneal dialysis patients, and ESRD patient without dialysis, which may confound the results of the study.

Among the cognitive dysfunction, memory deficiencies are regarded as a pivotal aspect interfering the self-management in dialysis ESRD patients, which greatly influenced the therapeutic regimen and dietary restriction (O’Lone et al., 2016). However, the memory deficiencies related domains in hemodialysis patients have not been fully understood. The California Verbal Learning Test-Second Edition (CVLT-II) is broadly used in measuring declarative verbal memory functions, consisting of episodic verbal learning and recall (Chepenik et al., 2012). To the best of our knowledge, no one has ever used the CVLT-II to evaluate the memory deficit in hemodialysis patients. More importantly, the relationship between spontaneous activity and verbal memory alterations has not yet been studied in hemodialysis patients.

In our study, we made a hypothesis that ESRD patients on hemodialysis may have altered spontaneous brain activity, which may account for the memory deficit in this population. To validate this hypothesis, resting state-functional MRI with ALFF methods was used to assess intrinsic brain activity in hemodialysis patients.

## Materials and Methods

### Subjects

This study was in line with the Helsinki Declaration and approved by the local Human Ethics Committee of Hainan General Hospital. All individuals were righthanded and they signed written informed consent before participating in the study.

Thirty-seven individuals (hemodialysis, *n* = 19; healthy controls, *n* = 18) were enrolled in this study. The hemodialysis patients were required to have chronic kidney disease with a disease duration of more than 3 months for all patients with ESRD and treated with regular hemodialysis for at least three months. Hemodialysis duration and etiology were extracted from patients’ medical records. The healthy volunteers were enrolled by advertisements.

Those met one of the following criteria will be excluded: (a) present of history of drug or alcohol addiction; (b) the obvious brain injuries on MR imaging; (c) history of or present psychiatric diseases; (d) other systemic diseases; (e) obvious visual or hearing abnormalities ; (f) previous history of organ transplantation; and (g) head motion exceeded 2.

All subjects were evaluated with a series of neuropsychological tests before MR examination: Montreal Cognitive Assessment (MoCA; Nasreddine et al., 2005); the number connection test type A (NCT-A), California Verbal Learning Test-II (CVLT-II), digit-span test (DS), digital symbol substitution test (DSST), line tracing test (LTT) and serial dotting test (SDT). MoCA could be used as a valuable tool to screen for mild cognitive impairment. The neuropsychological tests including NCT-A, DS, LTT, and SDT assess the domains of psychomotor speed, attention, and visual memory (Bajaj et al., 2009). The CVLT performance includes subscores for total immediate recall (trials 1–5, T1–5), short delay cued and free recall (SDCR, SDFR), and long delay cued and free recall (LDCR, LDFR).

All hemodialysis patients and healthy controls (HC) underwent biochemical tests to assess their renal function including creatinine, serum urea, uric acid, red blood cells (RBC), hemoglobin (Hb), hematocrit (Hct), phosphate (P) and calcium (Ca^2+^) before MR imaging.

### MRI Scanning

Resting-state fMRI data were obtained by using a 3-T MR scanner (TIM Skyra, Siemens Medical Solutions, Erlangen, Germany). Foam paddings were used to reduce head movement. They were told to maintain still, keep their eyes open but avoid thinking of anything during scanning. The parameters of T1 sequence are as follows: 20 axial sections; section thickness = 6 mm; intersection gap = 0.4 mm; in-plane resolution = 320 × 256; field of view (FOV) = 240 × 240 mm^2^; repetition time (TR) = 2,500 ms; echo time (TE) = 9 ms. T2-FlAIR images (20 axial sections; section thickness = 6 mm; in-plane resolution = 232 × 256; FOV = 240 × 240 mm^2^; flip angle = 150°) were acquired in all subjects to exclude any obvious lesions. Then, the parameters of high-resolution T1-weighted (T1WI) structural images were as follows: TR/TE = 2,530/2.98 ms, FOV = 256 × 256 mm, matrix size = 256 × 256, 192 sagittal slices with thickness of 1 mm. Finally, a gradient-echo echo-planar imaging (GRE-EPI) sequence was used to obtain functional sequence:TR/TE = 2,000/30 ms, FOV = 224 × 224 mm, matrix = 64 × 64, section thickness = 3.5 mm, 32 axial slices. The fMRI data of each candidate contains 240 brain volumes. The image acquisition time was 486 s.

### Image Processing

The functional imaging data preprocessing using the Data Processing Assistant for Resting-State fMRI toolbox^1^ (Yan et al., 2016). The first 10 time points of fMRI data were discarded for the signal equilibrium. Only 230 volumes were used for subsequent preprocess. First, slice timing and realignment were conducted. The Friston 24-parameter model (Friston et al., 1996) was regressed out to eliminate head motion impacts. Individual head translations, rotations, and the framewise displacement were calculated, and only one healthy control in this study was found to have excessive head motion. Second, the T1 structural image was co-registered to the functional image, then and then normalized to the standard Montreal Neurological Institute (MNI) space with Exponentiated Lie algebra (DARTEL) as in the study of Qi et al. (2019). Third, the white matter signal and cerebrospinal fluid signal were also regressed out. Lastly, the processed volume was normalized to the standard MNI space at 3 × 3 × 3 mm. The normalized data were smoothed with a 4-mm full width at half maximum.

ALFF was computed with the same method as described in the previous study (Qi et al., 2019). After bandpass filtered (0.01–0.1 Hz) and detrended, the time course was transformed to the frequency domain with Fast Fourier Transform. Thus, the power spectrum was counted and averaged it through 0.01–0.1 Hz at every voxel. The ALFF was defined as the averaged square root. The ALFF map was divided by the global mean ALFF value of each individual’s ALFF map (Zang et al., 2007).

### Statistical Analysis

The demographic and neuropsychological data were analyzed with SPSS version 21 (IBM Corp., Armonk, NY, USA). The ALFF group differences between hemodialysis and HC were calculated with an independent-sample *t*-test with SPM 12. Age, sex, and education were regressed out to reduce possible related confounding elements (Liu et al., 2014). Multiple comparisons were conducted (corrected *P* < 0.05) with the Gaussian random-field theory (GRF; Worsley et al., 2004) with voxel level set at *P* < 0.01 and cluster level set at *P* < 0.05.

To evaluate the association between ALFF value and neuropsychologic test scores, chemical results of patients on hemodialysis, the ALFF values were extracted from the cluster that showed statistical differences between the two groups in the patient groups. The extracted ALFF value was correlated with neuropsychologic test scores and chemical indices that showed a statistical difference between two groups by applying Spearman correlation analysis with a significant level of *P* < 0.05 (two-tail test, Bonferroni corrected).

## Results

The demographic, neuropsychological data, and clinical biochemical indices for patients and HC were demonstrated in Table 1. No statistical differences were found regarding sex, age, education level, NCT-A, SDFR, SDCR, LDCR, DS, Hct, and P between the two groups (all *P* > 0.05). Lower scores in MoCA, T1–5, LDFR, and DSST were found in hemodialysis patients relative to HC (*P* < 0.05). Hemodialysis patients had statistically lower levels in RBC and Hb, but higher levels in urea, creatinine, uric acid, and Ca^2+^ (all *P* < 0.05).

**Table 1:** Demographic and clinical data of hemodialysis patients and healthy controls.

	HD (*n* = 19)	HC (*n* = 17)	*P*-value
Gender (males/females)	6/13	9/8	0.194^a^
Age (year)	45.2 ± 6.7	41.8 ± 9.9	0.225^b^
Education (year)	10.2 ± 2.7	12.2 ± 3.0	0.076^c^
Dialysis duration (month)	86.2 ± 60.6	/	
Disease duration (month)	110.2 ± 60.6	/	
MoCA (score)	25.0 ± 3.2	27.1 ± 2.4	**0.033**^b^
NCT-A(s)	44.2 ± 32.9	42.6 ± 26.2	0.881^b^
T1–5 (score)	38.7 ± 11.3	47.8 ± 14.6	**0.044**^b^
SDFR (score)	8.7 ± 3.1	10.5 ± 3.7	0.127^b^
SDCR (score)	9.0 ± 3.0	11.1 ± 3.9	0.081^b^
LDFR (score)	8.5 ± 3.4	11.3 ± 3.7	**0.025**^b^
LDCR (score)	8.8 ± 3.1	10.9 ± 4.0	0.089^b^
DS (score)	12.5 ± 1.7	13.9 ± 3.1	0.105^b^
DSST (score)	36.0 ± 11.9	48.0 ± 18.0	**0.023**^b^
LTT(s)	55.4 ± 24.7	53.5 ± 18.3	0.803^b^
SDT(s)	44.7 ± 25.4	42.6 ± 13.6	0.770^b^
Urea (μmol/L)	22.1 ± 7.2	4.2 ± 1.0	**<0.001**^b^
Creatinine (μmol/L)	967.3 ± 209.1	66.0 ± 17.1	**<0.001**^b^
Uric acid (μmol/L)	429.6 ± 75.9	302.6 ± 84.8	**<0.001**^b^
RBC (×10^12^)	4.4 ± 1.0	4.9 ± 0.4	**0.040**^b^
Hb (g/L)	128.5 ± 24.3	145.3 ± 20.6	**0.033**^b^
Hct(%)	40.2 ± 8.0	44.3 ± 5.2	0.075^b^
P (mmol/L)	1.9 ± 0.6	1.3 ± 0.8	**0.022**^b^
Ca^2+^ (mg/L)	60.5 ± 9.2	56.5 ± 4.3	0.099^b^

*^a^*P*-value obtained with Chi-square test; ^b^*P*-value obtained with independent *t*-test for continuous variables; ^c^*P*-value obtained with independent *t*-test for continuous variables. Values are given as mean ± SD except for gender, which is presented as a number. HD, hemodialysis; HC, healthy control; MoCA, Montreal Cognitive Assessment; NCT-A, number connection test A; T1–5, Trial 1–5; CVLT-II, California Verbal Learning Test; SDFR, Short Delayed Free Recall; SDCR, Short Delayed Cue Recall; LDFR, Long Delayed Free Recall; LDCR, Long Delayed Cue Recall; DS, digit-span test; DSST, digital symbol substitution test; LTT, line tracing test; SDT, serial dotting test; RBC, red blood cells; Hb, hemoglobin; Hct, hematocrit; Ca^2+^, calcium; P, phosphorus*.

### Group Differences in ALFF

**Figure 1:**
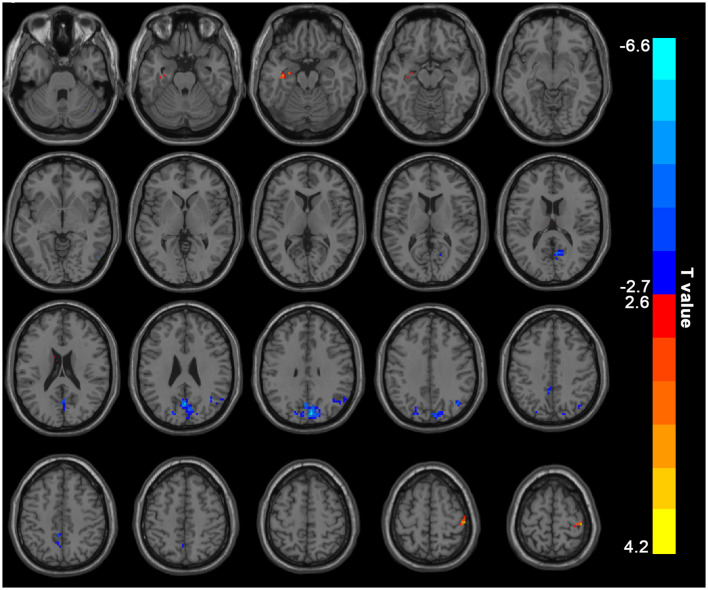
Mean ALFF maps show differences between ESRD patients on hemodialysis and HC (*p* < 0.05, GRF corrected). The hemodialysis ESRD group showed significantly lower mean ALFF values in the precuneus, cuneus, angular gyrus/inferior parietal lobule, but increased mean ALFF values in the left parahippocampal gyrus/hippocampus, right postcentral/precentral gyrus relative to both the HC group. ALFF, amplitude of low-frequency fluctuation; HC, healthy control subjects; ESRD, end-stage renal disease. The color bar represents *t*-values from the two-sample *t*-test. Colors in red and blue respectively indicate a significant increase and decrease in the two-sample *t*-test.

The hemodialysis patients had decreased ALFF in the bilateral precuneus/cuneus, right angular gyrus/inferior parietal lobule when compared to the HC (Figure 1, Table 2). Hemodialysis patients displayed increased ALFF in the left parahippocampus/hippocampus and right precentral/postcentral gyrus relative to HC (Figure 1, Table 2).

**Table 2:** Brain regions showing differences in mALFF between the hemodialysis and HC groups.

Brain region	Voxel	Peak *T* score	MNI coordinates		
			x	y	z
Precuneus/Cuneus (bilateral)	195	−6.5649	6	−81	33
Right angular gyrus/inferior parietal lobule	40	−4.46	39	−69	39
Left Parahippocampus Gyrus/Hippocampus	15	3.7825	−36	−15	−18
Right PreCG/PoCG	32	4.1637	42	−27	66

*HC, healthy control; MNI, Montreal Neurological Institute; PreCG, precentral gyrus; PoCG, postcentral gyrus*.

### Correlation Analysis Results

**Figure 2:**
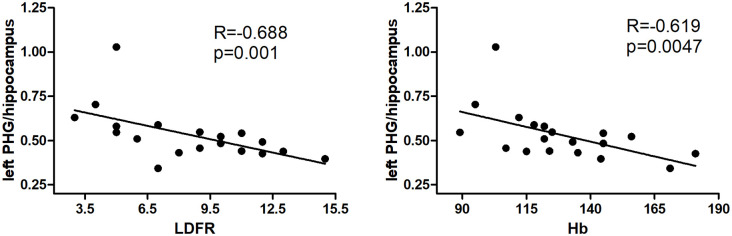
Results of correlation analyses between the left precuneus and parahippocampus/hippocampus ALFF values and hemodialysis clinical indices. The LDFR and Hb are negative with ALFF in the left PHG/hippocampus (*P* < 0.05, Bonferroni corrected). PHG, parahippocampal gyrus; LDFR, Long-Delayed Free Recall; Hb, hemoglobin.

ALFF values of the left parahippocampus/hippocampus showed a negative correlation with LDFR score, and hemoglobin in hemodialysis patients (*R* = −0.688, *p* = 0.001; *R* = −0.619, *p* = 0.0047; Figure 2).

## Discussion

This study revealed abnormal ALFF in some DMN regions in hemodialysis patients. The hemodialysis group displayed decreased spontaneous brain activity in the precuneus/cuneus and angular gyrus, but increased activity in the parahippocampus/hippocampus compared with HC, which is associated with neuropsychological and clinical indices. Our results suggested that strategies should be taken for hemodialysis patients to improve their cognitive function.

The present study found decreased ALFF values in the DMN regions in hemodialysis patients. The DMN is involved in multiple brain functions including memory, visuality, and auditory attention, motor performance, and language processing (Buckner et al., 2008; Kim, 2012). Impairment of these neurocognitive areas has been found to lead to significant impairment in ESRD patients irrespective of whether they are receiving hemodialysis or peritoneal dialysis (Luo et al., 2016; Li et al., 2018). As a pivotal component of the DMN, the precuneus is closely associated with integrated tasks such as keeping wakefulness and the regulation of visual-spatial episodic memory (Buckner et al., 2009). The inferior parietal lobe is a key component of the DMN (Fransson and Marrelec, 2008). Decreased ALFF in the right inferior parietal lobe may imply a disturbed memory function in patients with ESRD. Our results are fitted well with the results of previous studies (Liang et al., 2013; Ni et al., 2014; Luo et al., 2016). Luo et al. (2016) found that patients with ESRD on peritoneal dialysis group showed further reduced ALFF values mainly in the DMN regions including left inferior parietal lobe and left precuneus than did those in the nondialysis group with ESRD.

Importantly, aberrant brain activity in some regions was found to be correlated with a long-delayed free recall score of the CVLT-II in the hemodialysis group. CVLT is widely used to assess declarative verbal memory functions(Chepenik et al., 2012). The result of our study is identical to the result by Li et al. (2018), who found that the ESRD group showed poor performance in the immediate recall total score, short-term delayed recall score, and long-term delayed recall score. Free-recall might be an important aspect of memory dysfunction in hemodialysis patients. More importantly, our study found that ALFF values in the left parahippocampus/hippocampus negatively correlated with long delay free recall score. Parahippocampus /hippocampus has been found to play a pivotal role in memory functions, learning, and cognition (Braun, 2011; Zeidman and Maguire, 2016), especially memorizing facts and events. Increased ALFF values in the parahippocampus/hippocampus may indicate compensation in the memory function to maintain a normal function. Similarly, higher regional homogeneity was also found in the previous study (Li et al., 2018). The hyperactivity of the left hippocampus might be associated with hyper-perfusion in this region found by Cheng et al. (2019) in a recent study. Together with the findings described above, we may deduce that the abnormality in the parahippocampus/hippocampus may be involved in the memory deficits in hemodialysis ESRD patients.

The urea, creatinine, and uric acid were significantly higher in hemodialysis patients. Unexpectedly, urea, creatinine, and uric acid levels showed no correlation with ALFF values in the brain after multiple comparisons corrected. The uremic toxins including urea and serum creatinine were specifically high in ESRD patients because of renal failure (Owen et al., 1993). Previous studies have found the abnormal spontaneous activity correlated with serum creatinine and urea levels (Liang et al., 2013; Chen et al., 2015). The small sample size and different methodology may account for the inconsistency. Urea had been reported to play an important part in the brain edema development in dialysis disequilibrium syndrome in a rat study (Galons et al., 1996). Hemoglobin levels were negative with the ALFF values in the left parahippocampus/hippocampus. Luo et al. (2016) also found that elevated serum urea and decreased hemoglobin levels have an influence on intrinsic brain activity in peritoneal dialysis patients with ESRD. Increasing evidence has suggested that anemia in patients with ESRD might lead to cerebral hyper-perfusion, which finally results in cognitive dysfunction (Jiang et al., 2016; Cheng et al., 2019). The neurophysiological performance could be improved after anemia treatment (Kambova, 1998; Pickett et al., 1999; Kurella Tamura and Yaffe, 2011). Therefore, our study further supports the notion of anemia treatment in hemodialysis patients are beneficial for cognitive improvement.

The main strengths of the study are as follows. We used relatively comprehensive neuropsychological tests in consultation other than applying a simple screening test for assessing cognition. Besides, we also evaluated the blood chemical indices in HC.

This study had several limitations. First, the sample size of our study is relatively small, therefore, our results should be considered preliminary. Further studies with a larger sample size should be considered in the future. Second, this is a preliminary study, we did not consider etiology. How ESRD etiology affects brain activity in hemodialysis should be further explored. Third, the study was cross-sectional designed. A longitudinal study focusing on the changes in ESRD patients before and after hemodialysis should be considered in the future. Fourth, the influence of blood pressure on cerebral function in patients on hemodialysis was not included in this study.

In conclusion, we found that hemodialysis patients had abnormal intrinsic brain activity in the DMN regions including the precuneus and parahippocampus/hippocampus. These findings provided further evidence to support the notion that the DMN regions are involved in the cognition pathophysiology in hemodialysis.

## Data Availability Statement

The datasets in this study are not available currently because the present data is part of an ongoing longitudinal study and most data are still in collection and ought to be protected. Reasonable requests to obtain the data could be emailed to FC, fenger0802@163.com.

## Ethics Statement

The studies involving human subjects were reviewed and approved by the Medical Research Ethics Committee of Hainan General Hospital in accordance with the Helsinki Declaration. Written informed consent was acquired by all the participants before the study.

## Author Contributions

HC, JQ, and FC contributed to the conception and design of the study. HC, JQ, QF, and FC performed the statistical analysis and wrote the manuscript. HC, JQ, and QF performed the experiments. All authors contributed to manuscript revision and read and approved the submitted version.

## Conflict of Interest

The authors declare that the research was conducted in the absence of any commercial or financial relationships that could be construed as a potential conflict of interest.

## Footnotes

^1^http://www.restfmri.net/forum/DPARSF
